# Comparative study of quality of life 9 months post-COVID-19 infection with SARS-CoV-2 of varying degrees of severity: impact of hospitalization vs. outpatient treatment

**DOI:** 10.3389/fsoc.2023.1143561

**Published:** 2023-05-16

**Authors:** Olga Maslova, Tatiana Vladimirova, Arseny Videnin, Saikat Gochhait, Vasily Pyatin

**Affiliations:** ^1^Neurosociology Laboratory, Neurosciences Research Institute, Samara State Medical University, Samara, Russia; ^2^Department of Otorhinolaryngology, Samara State Medical University, Samara, Russia; ^3^Institute of Clinical Medicine, Samara State Medical University, Samara, Russia; ^4^Symbiosis International (Deemed University), Pune, India

**Keywords:** post-COVID-19 conditions, patients with post-COVID-19, SARS-CoV-2, health-related quality of life, patient-reported outcome

## Abstract

**Purpose:**

This experimental study was conducted during the post-COVID-19 period to investigate the relationship between the quality of life 9 months after and the severity of the SARS-CoV-2 infection in two scenarios: hospitalization (with/without medical oxygen) and outpatient treatment.

**Methods:**

We employed the EQ-5D-5L Quality of Life tests and the PSQI as a survey to evaluate respondents' quality of life 9 months after a previous SARS-CoV-2 infection of varying severity.

**Results:**

We identified a clear difference in the quality of life of respondents, as measured on the 100-point scale of the EQ-5D-5L test, which was significantly lower 9 months after a previous SARS-CoV-2 infection for Group 1 (*n* = 14), respondents who had received medical attention for SARS-CoV-2 infection in a hospital with oxygen treatment, compared to those with the SARS-CoV-2 infection who were treated without oxygen treatment (Group 2) (*n* = 12) and those who were treated on an outpatient basis (Group 3) (*n* = 13) (H = 7.08 *p* = 0.029). There were no intergroup differences in quality of life indicators between hospitalized patients (Group 2) and groups 1 and 3. PSQI survey results showed that “mobility,” “self-care,” “daily activities,” “pain/discomfort,” and “anxiety/ depression” did not differ significantly between the groups, indicating that these factors were not associated with the severity of the SARS-CoV-2 infection. On the contrary, the respondents demonstrated significant inter-group differences (H = 7.51 *p* = 0.023) and the interdependence of respiratory difficulties with the severity of clinically diagnosed SARS-CoV-2 infection. This study also demonstrated significant differences in the values of sleep duration, sleep disorders, and daytime sleepiness indicators between the three groups of respondents, which indicate the influence of the severity of the infection. The PSQI test results revealed significant differences in “bedtime” (H = 6.00 *p* = 0.050) and “wake-up time” (H = 11.17 *p* = 0.004) between Groups 1 and 3 of respondents. At 9 months after COVID-19, respondents in Group 1 went to bed at a later time (p*p* = 0.02727) and woke up later (*p* = 0.003) than the respondents in Group 3.

**Conclusion:**

This study is the first of its kind in the current literature to report on the quality of life of respondents 9 months after being diagnosed with COVID-19 and to draw comparisons between cohorts of hospitalized patients who were treated with medical oxygen vs. the cohorts of outpatient patients. The study's findings regarding post-COVID-19 quality of life indicators and their correlation with the severity of the SARS-CoV-2 infection can be used to categorize patients for targeted post-COVID-19 rehabilitation programs.

## Introduction

The continuation or persistence of symptoms after the acute phase of SARS-CoV-2 infection is commonly known as long COVID-19 or post-COVID-19. These symptoms can range from general (e.g., fever, myalgia, fatigue, and tiredness) to neurological, psychological, and cognitive symptoms (Amdal et al., 2021; Belopasov et al., 2021; Pazukhina et al., 2022). In most of the published studies, symptoms of post-COVID-19 have been observed in patients for up to 6 months after receiving treatment in a hospital or in an outpatient setting (Lopez-Leon et al., 2021; Michelen et al., 2021; Nasserie et al., 2021). It was found that the prevalence of post-COVID-19 symptoms varies significantly between hospitalized and non-hospitalized patients (Peghin et al., 2021). Thus, post-COVID-19 symptoms were observed in 54% of hospitalized patients and in 34% of non-hospitalized patients (Chen et al., 2022). Prolongation of post-COVID-19 symptoms among hospitalized and non-hospitalized patients has been reported to persist for a long period of time, that is, ranging from up to 3–6 months (Peghin et al., 2021; Sivan M, et al., 2022) to even up to 2 years after the SARS-CoV-2 infection (Fernández-de-las-Peñas et al., 2022). Moreover, Sivan et al. (2022) reported for the first time on the phenotypes of symptom severity in a cohort of people who were mostly not hospitalized. With regard to the clinical symptoms of the disease in the SARS-CoV-2 infection, the presence of post-COVID-19 symptoms was significantly associated with the number of symptoms at the beginning of the disease and the degree of its severity that requires hospitalization in the intensive care unit (ICU) (Del Rio et al., 2020; Carvalho-Schneider et al., 2021; Pérez-González et al., 2022).

In general, it can be stated that there are relatively few direct comparisons of post-COVID-19 symptoms among hospitalized and non-hospitalized respondents in the literature compared to the studies on SARS-CoV-2-infected patients during hospitalization. For example, a recent review provided five references to studies directly comparing the differences and prevalence of post-COVID-19 symptoms between previously hospitalized and non-hospitalized subjects. However, observations were from follow-up of only 3 months post-COVID-19 (Van Kessel et al., 2022).

There are no studies in the literature on the new paradigm of comparisons, namely, differences in the quality of life post-COVID-19 among hospitalized patients who were prescribed medical oxygen during the acute phase of SARS-CoV-2 infection and among hospitalized patients who were treated without oxygen therapy. However, acute hypoxic respiratory failure is the most common complication that occurs in 60–70% of patients, and patients that developed this complication were admitted to the ICU (Phua et al., 2020). Therefore, medical oxygen is a critical element in the treatment of patients with COVID-19 (Saadatmand et al., 2022), as active oxygen therapy to treat hypoxia is important for positive patient outcomes. Moreover, patients who survived hospitalization due to COVID-19 received additional oxygen treatment at home to treat persistent hypoxemia after discharge (Kaul et al., 2022). Consequently, to date, no long-term comparative study on the quality of life in post-COVID-19 subjects with different disease severity at hospital admission has been conducted. In the active phase of SARS-CoV-2 infection, the quality of life of the patients was the object of analysis in publications (Amdal et al., 2021), but it is not clear to what extent the quality of life and health indicators of the active phase of the disease was prolonged in the post-COVID-19 period. Therefore, a comparative study of the quality of life after 6 months of post-COVID-19 of respondents who have experienced SARS-CoV-2 infection with varying degrees of severity during hospitalization and of those who received outpatient treatment is relevant.

This study aimed to investigate the impact of different degrees of severity of the SARS-CoV-2 infection during hospitalization on the quality of life of respondents 9 months post-COVID-19. Specifically, the study examined the quality of life of hospitalized respondents who received medical oxygen treatment vs. those who did not. It is critical to understand that the relevance of the question lies in the consequences of the infection if the person becomes a survivor after completing medical treatment (Pomara et al., 2020). SARS-CoV-2 infection has several consequences (Amdal et al., 2021; Hayes et al., 2021). Moreover, this study examined a new aspect of the management of COVID-19 survivors, namely the post-COVID-19 quality of life of those who were hospitalized due to the COVID-19 condition in the ICU as well as that of those who were hospitalized but were not treated in an intensive care setting.

The COVID-19 pandemic has adversely affected the population's quality of life in all spheres of life, causing a negative impact on their overall wellbeing. In several studies, it has been found that people with a coronavirus infection experience significant physical and emotional impacts on their lives, including their social functioning, which is markedly affected. Some of these consequences can last for 3 months or more, with varying degrees of severity (Poude et al., 2021). Furthermore, sleep disorders have been associated with patients who have been infected with SARS-CoV-2 as a result of the infection, and it has been documented that these disorders can worsen the severity of the infection, reducing the quality of life of the patient in the process. Tedjasukmana et al. (2023) conducted an online survey of the condition following COVID-19 in different countries, which found that 78.58% of respondents had sleep disorders, including insomnia, sleep breathing disorders, hypersomnolence, sleep-wake circadian rhythm disorders, parasomnia, and sleep-related movements. As a result, several SF-36 quality-of-life parameters were statistically significant positive predictors of moderate to severe insomnia in the SF-36 scale. A statistically significant positive correlation was found between various areas of the SF-36 quality of life questionnaire and the Pittsburgh Sleep Quality Index (PSQI) when assessing the global assessment of conditions after COVID-19 using the PSQI. The relationship between sleep disorders and mental health disorders is closely interconnected, highlighting the urgent need for intervention strategies to prevent mental health disorders, including sleep disorders, and improve rehabilitation and patients' quality of life after COVID-19.

Several questionnaires and scales are commonly used to assess the quality of life of patients (Hawthorne et al., 2001), including the following tools: the 36-item Short Form (SF-36) survey (RAND Corporation, 2022) and the Centers for Disease Control and Prevention's (CDC) 14-item Health Related Quality of Life (CDC HRQOL-14) (CDC HRQOL−14, 2022); in terms of the SF-36, it can be described as a short form questionnaire.

In 2009, the EuroQol Group introduced a five-level EQ-5D [EuroQol Group (EQ-5, 2019)] to improve its psychometric properties and facilitate its widespread use for patients with COVID-19 after discharge worldwide (Feng et al., 2021; Nandasena et al., 2022).

This study aimed to determine whether sleep disturbances and quality of life were significantly improved in patients with COVID-19 9 months after discharge from the hospital.

## Materials and methods

The study was conducted between September 2021 and October 2021 with patients aged 18 years and older who were diagnosed with COVID-19 and who were treated at the clinics of Samara State Medical University in 2021 and had successfully recovered. The patients were followed up 9 months after their discharge from the hospital post-recovery. According to the Helsinki Declaration of Ethical Standards, the study was conducted in accordance with Samara State Medical University's ethical standards, as approved by its ethics committee (Protocol No. 196). All survey respondents provided informed consent to participate in the study before they were included in it.

### Inclusion criteria

There are a number of inclusion criteria that needed to be met. These criteria included (1) being at least 18 years of age, (2) having been diagnosed with COVID-19 and recovered, (3) having been treated at SamSMU clinics (either as an inpatient or outpatient), and (4) being willing to provide informed consent to participate in the survey. Of 123 discharged patients, 39 of them met these selection criteria and were included in the study, comprising 15 men and 24 women (Table 1).

**Table 1:** Description of groups.

**Characteristics**	**Mean** ±**standard error of mean**			**Mann-Whitney *U* Test, p_0_**	**Kruskal-Wallis test**
	**Gr.1**	**Gr.2**	**Gr.3**		
Number of respondents	14	12	13	-	-
Age of respondents	60.0 ± 2.1	54.2 ± 1.5	58.5 ± 1.0	Gr.1 and Gr.2–0,076 Gr.1 and Gr.3–0,645 Gr.2 and Gr.3–0,073	H = 5,68 *p* = 0,058
The number of days spent by the respondent in the hospital during the treatment of COVID-19	15.1 ± 1.9	13.2 ± 0.9	0.0 ± 0.0	Gr.1 and Gr.2–1,000	-
On what day of the COVID-19 disease was the respondent admitted to the hospital	7.6 ± 0.9	8.7 ± 1.7	0.0 ± 0.0	Gr.1 and Gr.2–0,526	-

### Patients

A total of 39 adult patients who had acute respiratory failure participated in this study after their treatment while staying in the hospital and after treatment in an outpatient setting. The study was conducted between September and October of 2021, nearly 9 months after the acute phase of coronavirus infection associated with SARS-CoV-2 had ended. Participants were recruited online from a database of patients diagnosed with COVID-19 provided by the Otorhinolaryngology Department of Samara State Medical University. The results of the survey led to the formation of three groups of respondents. The first group consisted of hospitalized respondents who received medical oxygen during the treatment period. The second group of respondents had a history of hospitalization and treatment without medical oxygen. The third group of respondents had a history of outpatient treatment during the acute period of the SARS-CoV-2 infection.

### Period of infection

The period of infection was defined as the number of days spent by the respondent in the hospital during the treatment of COVID-19. In Group 1, the average length of stay was 15.1 ± 1.9 days, while in Group 2, it was 13.2 ± 0.9 days (Table 1).

### Data collection and measures

To prevent the spread of COVID-19 and comply with the ethical protocol of the ethical board, data collection was conducted online and via a telephone-based survey. Data were collected using a medical database and two questionnaires. The first section comprised demographic questions related to age and gender and to hospitalization status in terms of the start date of staying in the intensive care unit, the discharge date from the intensive care unit, the number of days spent in the intensive care unit, and the first day of hospitalization from the beginning of the illness (Socio-Demographic Questions). These questions were based on the demographics and hospitalization status of a database provided by the Otorhinolaryngology Department of Samara State Medical University.

The second session involved the administration of the official Russian version of the Quality of Life Questionnaire (EQ-5D-5L). In the third session, the participants completed the Pittsburgh Sleep Quality Index (PSQI) (Buysse et al., 1989; Luca et al., 2015). The participants were interviewed by three physicians from the Otorhinolaryngology Department of Samara State Medical University.

The EQ-5D-5L questionnaire consists of two sections: the descriptive system and the visual analog scale (EQ VAS). The EQ-5D-3L collects information on a respondent's quality of life in the form of a health profile described by three levels of problem expression in five components (mobility, self-care, usual activities, pain/discomfort, and anxiety/depression). The EQ-VAS is a visual analog scale that is used to assess a respondent's self-rated health status. The EQ-5D questionnaire also yields an EQ-5D index score, which is a measure of overall health status (Feng et al., 2014; Karimi and Brazier, 2016).

The EQ-5D-5L descriptive system consists of five dimensions as follows: mobility, self-care, usual activities, pain/discomfort, and anxiety/depression. Each dimension has five response levels: Level 1, “no problems;” Level 2, “slight;” Level 3, “moderate;” Level 4, “severe;” and Level 5, “extreme problems”.

The respondents were asked to indicate their health state by marking the box that corresponded to the most appropriate statement in each of the five dimensions. The EQ-5D-5L also includes a visual analog scale (EQ-VAS) rated on a scale of 0 to 100 mm, providing a single global rating of self-perceived health. The data collected using the EQ-5D-5L are presented in a descriptive system as a health profile. The results of the EQ VAS are presented as a measure of overall self-rated health status.

The Pittsburgh Sleep Quality Index (PSQI) is a widely used 19-item self-reported questionnaire for measuring sleep disturbances and healthy sleep [Buysse et al., 1989; Luca et al., 2015].

The PSQI includes seven clinically derived domains of sleep difficulties: sleep quality, sleep latency, sleep duration, habitual sleep efficiency, sleep disturbances, use of sleeping medications, and daytime dysfunction. Each domain score was calculated based on the participant's response to specific items, most of which were presented on a 0–3 Likert-type scale (with higher scores indicating poorer sleep quality). These sleep domains were combined into a single PSQI sleep quality factor, with a higher score indicating worse sleep quality. In addition to the global PSCI factor, a validated three-factor model of the PSQI was proposed to assess disturbances in three separate factors of subjective sleep reports: sleep efficiency, perceived sleep quality, and daily disturbances.

### Statistical analysis

Statistical data processing was performed in the STATISTICA 12 program. The normality of the distribution was checked according to the Shapiro–Wilk, Kolmogorov–Smirnov, and Lilliefors criteria. Most of the studied parameters were characterized by a different distribution from the normal one. The Mann–Whitney test was used to compare individual groups with each other. The Kruskal–Wallis test was used to compare all three groups. Closed-ended questions were visually presented as pie charts with a sector for each answer option. The value corresponded to the number of participants who chose this answer option. Integrative parameters were visually represented in the form of boxplot diagrams, where the upper and lower borders of the shaded rectangle indicate the corresponding quartiles, the horizontal line within the rectangle indicates the median, the cross indicates the arithmetic mean, the outflow lines indicate the maximum and minimum values and the horizontal line between the rectangles indicates the presence of statistically significant differences.

## Results

Three groups of patients who survived COVID-19 were included in the study. The first group (Gr. 1, *n* = 14, age 60.0 ± 2.1) consisted of six men and eight women, with a total of 14 individuals. This group consisted of patients who were treated in hospitals with medical oxygen for the SARS-CoV-2 infection and were included in this study. A total of 42.9% of the participants in the study were men, while 57.1% of them were women. The second group (Gr.2) included 12 patients with an infection of SARS-CoV-2 who were treated without medical oxygen. Men and women were represented equally in the group, with 58.3% of men and 41.7% of women. In the third group (Gr.3, *n* = 13, average age 58.5 =1.0), two men and 11 women were included among the 13 participants. Those who were treated for COVID-19 as outpatients were included in this study. Among the men and women in the group, the percentages of men and women were 15.4% and 84.6%, respectively. The ages of the group members did not differ significantly from each other.

**Figure 1:**
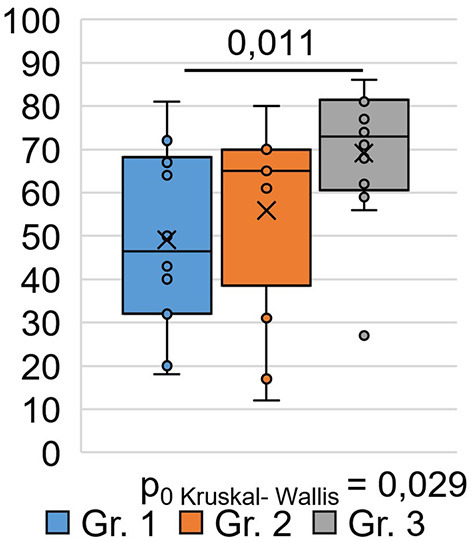
EQ-5D-5L, current subjective assessment of the condition.

A sociological survey was conducted via phone 9 months after the respondents were discharged from the hospital and who visited the hospital during the post-COVID-19 period for follow-up as outpatients. The EQ-5D-5L questionnaire was used to study respondents' quality of life at the time of their current state, which was 9 months after their recovery from COVID-19. An analysis of respondents ' responses to questions in the EQ-5D-5L questionnaire among the different groups (Table 2) showed that significant differences between the groups (H = 7.08 *p* = 0.029) occurred only in responses to the question (Figure 1), where the respondents had to describe their subjective state using a number on a 100-point scale.

**Table 2:** EQ-5D test results.

**Question**	**Response options**	**Responses**			**Mann-Whitney U Test, p_0_**	**Kruskal- Wallis**
		**Gr.1**	**Gr.2**	**Gr.3**		
Mobility	I have no difficulty walking	57.1%	75.0%	92.3%	Gr1 and Gr2–0,662 Gr1 and Gr3–0,126 Gr2 and Gr3–0,446	H = 3,83 *p* = 0,147
	I have some difficulty walking	42.9%	8.3%	7.7%		
	I'm bedridden	0.0%	16.7%	0.0%		
Self-care	I have no difficulty taking care of myself	71.4%	91.7%	100.0%	Gr1 and Gr2–0,382 Gr1 and Gr3–0,216 Gr2 and Gr3–0,744	H = 5,18 *p* = 0,075
	I have some difficulty washing or dressing	21.4%	8.3%	0.0%		
	I am not able to wash or dress myself	7.1%	0.0%	0.0%		
Daily activities	I am not experiencing difficulties	64.3%	75.0%	100.0%	Gr1 and Gr2–0,758 Gr1 and Gr3–0,120 Gr2 and Gr3–0,301	H = 5,17 *p* = 0,076
	I'm having some difficulties	35.7%	16.7%	0.0%		
	I am not able to do my usual daily activities	0.0%	8.3%	0.0%		
Pain / Discom-fort	I don't feel any pain or discomfort	78.6%	83.3%	92.3%	Gr1 and Gr2–0,857 Gr1 and Gr3–0,560 Gr2 and Gr3–0,724	H = 0,97 *p* = 0,615
	I am experiencing moderate pain or discomfort	21.4%	16.7%	7.7%		
	I am experiencing extremely severe pain or discomfort	0.0%	0.0%	0.0%		
Anxiety / Depression	I don't experience anxiety or depression	71.4%	91.7%	100.0%	Gr1 and Gr2–0,368 Gr1 and Gr3–0,216 Gr2 and Gr3–0,744	H = 5,26 *p* = 0,072
	I am experiencing moderate anxiety or depression	14.3%	8.3%	0.0%		
	I am experiencing extremely severe anxiety or depression	14.3%	0.0%	0.0%		
Condition today: subjective assessment on a 100-point scale, where 0 is disgusting, and 100 is fine	0–25	0.0%	0.0%	0.0%	Gr1 and Gr2–0,537 Gr1 and Gr3–0,011 Gr2 and Gr3–0,073	H = 7,08 *p* = 0,029
	26–50	57.1%	25.0%	7.7%		
	51–75	35.7%	66.7%	53.8%		
	76–100	7.1%	8.3%	38.5%		
	Mean	49.07	55.83	69.15		

Moreover, 0 points corresponded to a highly negative assessment of the quality of life 9 months after COVID-19, and 100 points corresponded to a positive assessment of the quality of life after 9 months post-COVID-19. As shown in Figure 1, the subjective state of respondents in Group 1 was significantly (0.011) worse than that of respondents in Group 3: the average score on a 100-point scale was 49.1 ±5.4 in Group 1 and 69.2 ±4.4 in Group 3. Here, 0 points corresponded to a highly negative quality of life score at 9 months post-COVID-19, and 100 points corresponded to a positive quality of life score at 9 months post-COVID-19. As shown in Figure 1, the subjective condition of respondents in Group 1 was significantly (0.011) worse than that of respondents in Group 3. The average score on a 100-point scale was 49.1 ±5.4 in Group 1 and 69.2 ±4.4 in Group 3. There was no significant difference in the EQ-VAS rating of respondents in Groups 1 and 2 compared with respondents in Groups 2 and 3 (Figure 1).

**Figure 2:**
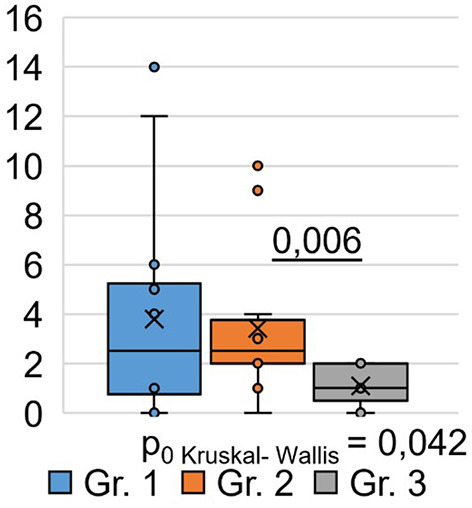
PSQI, composite score.

Using the Pittsburgh Sleep Quality Questionnaire (PSQI), we designed the study to analyze inter-group differences among respondents at 9 months post-COVID-19. We found that the total score based on the results (Table 3) of the PSQI (*p* = 0.042) differed significantly between the groups (Figure 2). Thus, in Group 1, the total score averaged 3.79 ± 1.18, while in Group 2, it was 3.42 ± 0.87. In Group 3, the average total score was 1.08 ± 0.21.

**Table 3:** Scores of the Pittsburgh Sleep Quality Index (PSQI).

**Component**	**Average score for the group Mean** ±**standard error of mean**			**Mann-Whitney** ***U*** **Test**, ***p***_**0**_			**Kruskal-Wallis test**	
	**Gr.1**	**Gr.2**	**Gr.3**	**Gr1 and Gr2**	**Gr1 and Gr3**	**Gr2 and Gr3**	**H**	*p*_0_
Subjective sleep quality	0.79 ± 0.28	0.83 ± 0.17	0.54 ± 0.14	0.471	0.884	0.289	1.45	0.484
Sleep latency	0.36 ± 0.13	0.25 ± 0.18	0.08 ± 0.08	0.504	0.225	0.703	3.05	0.217
Sleep duration	0.57 ± 0.25	0.08 ± 0.08	0.00 ± 0.00	0.227	0.120	0.744	**7.25**	**0.027**
Sleep efficiency	0.43 ± 0.25	0.17 ± 0.11	0.08 ± 0.08	0.777	0.528	0.724	1.17	0.559
Sleep disturbance	0.71 ± 0.19	1.08 ± 0.23	0.31 ± 0.13	0.292	0.182	**0.016**	**7.29**	**0.026**
Use of sleep medication	0.43 ± 0.25	0.42 ± 0.26	0.00 ± 0.00	0.939	0.357	0.301	3.46	0.177
Daytime sleepiness	0.50 ± 0.20	0.58 ± 0.26	0.00 ± 0.00	0.877	0.120	0.082	**6.50**	**0.039**
Composite score	3.79 ± 1.18	3.42 ± 0.87	1.08 ± 0.21	0.857	0.174	**0.006**	**6.33**	**0.042**

Consequently, the quality of sleep for the respondents in Groups 1 and 2, who were hospitalized for COVID-19, remained significantly worse than that of the respondents in Group 3, who did not require hospitalization, even 9 months after their recovery. Analysis of the obtained data from the PSQI showed differences in the degree of influence of the severity of the SARS-CoV-2 infection on different components of sleep quality (Table 3).

Thus, the values of sleep duration, sleep disorders, and daytime sleepiness significantly differed between the three groups of respondents, which indicates that they are susceptible to the influence of a previous infection, SARS-CoV-2, affecting these components of sleep quality. The indicators “subjective sleep quality,” “time to sleep onset,” “sleep efficiency,” and “frequency of taking sleeping pills” did not show significant intergroup differences. This indicates that these sleep quality indicators are not affected by the severity of the SARS-CoV-2 infection in the studied respondents. However, analysis of respondents' responses to individual PSQI questions (Table 3) demonstrated the following significant differences.

**Figure 3:**
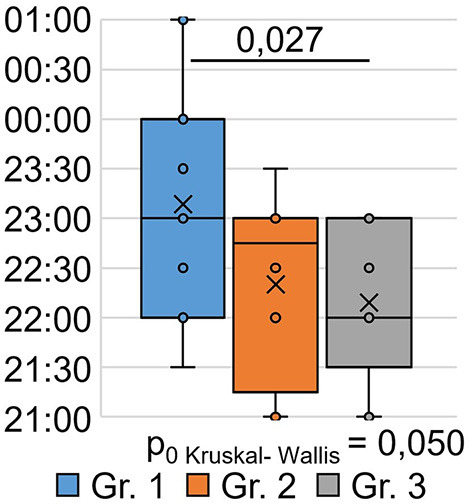
PSQI, when do respondents usually go to bed?

The indicator “bedtime” (Figure 3) significantly differed between the groups (H = 6.00 *p* = 0.050): patients who were treated for SARS-CoV-2 infection in an oxygen-supported hospital (group 1) went to bed at a later time after 9 months (*p* = 0.027) than the respondents in Group 3 who were treated for SARS-CoV-2 infection on an outpatient basis.

**Figure 4:**
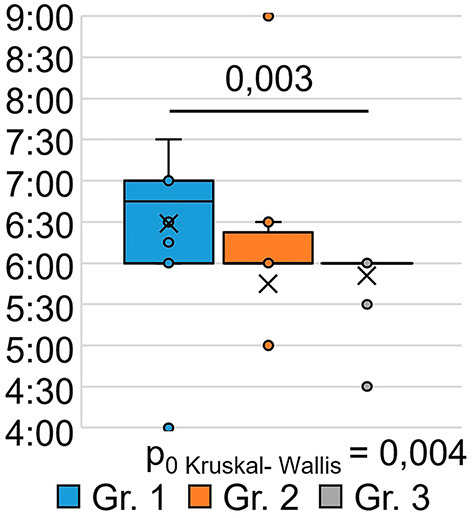
PSQI, when respondents usually get up in the morning.

**Figure 5:**
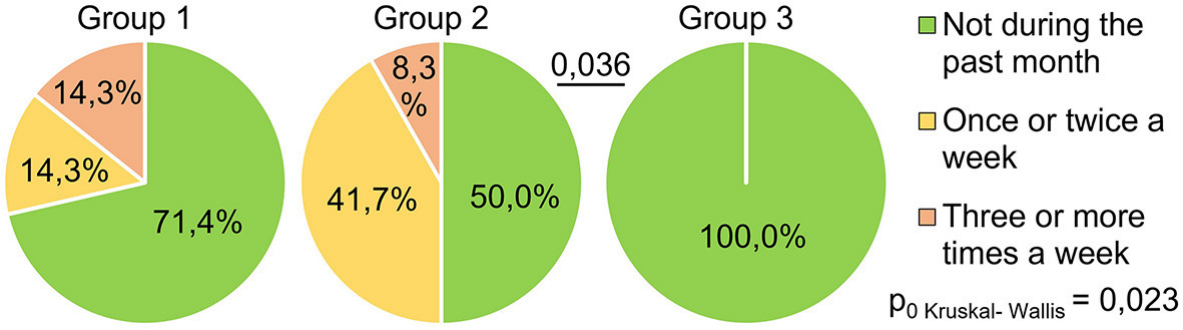
PSQI, how often during the past month did respondents have problems sleeping because they could not breathe comfortably?

The indicator “get up time” (Figure 4) also significantly differed between the groups (H = 11.17 *p* = 0.004): the respondents of Group 1 got up later than those of Group 3 (*p* = 0.003). This may indicate that the SARS-CoV-2 infection in its severe form (group 1) causes a long-term violation of circadian biorhythms in such respondents. Answers to the question “How often have you been unable to breathe freely?” (Figure 5) showed significant intergroup differences (H = 7.51 *p* = 0.023). In Group 1, 28.6% of respondents experienced similar breathing difficulties on average once a week or more. In Group 2, breathing difficulties occurred in 50% of respondents, and in Group 3, none of the respondents experienced this problem in the last month.

In addition to the data obtained as a result of analyzing the answers to the above questions, there were significant differences between all groups (Kruskal–Wallis test) in the answers to the questions of how often respondents felt that they were experience hot flashes and how often respondents had bad dreams. Moreover, there were no significant differences between individual groups (Mann–Whitney U Test) in these indicators.

**Figure 6:**
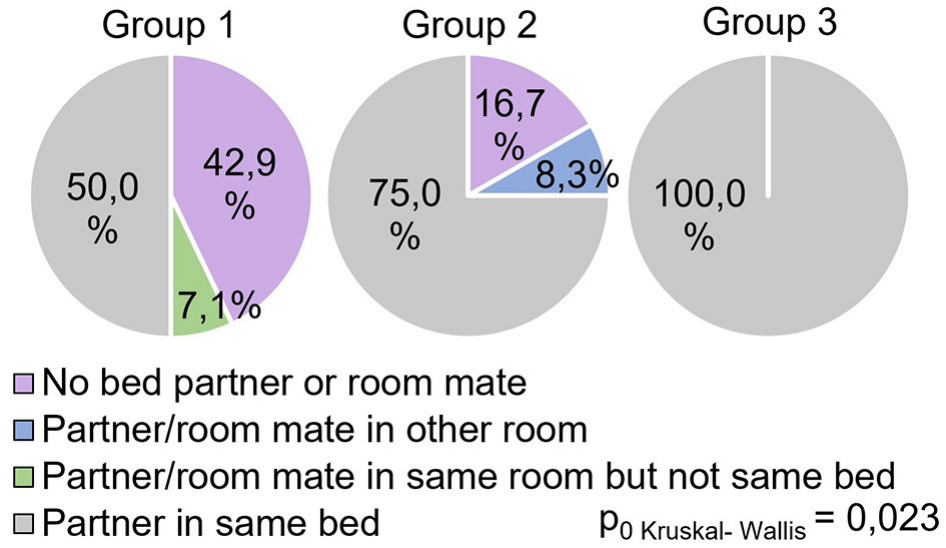
PSQI, do respondents have a bed partner or roommate?

The study showed significant differences between groups (H = 8.69 *p* = 0.013) in the answers to the question “Do respondents share the same bed with a partner during sleep?” (Figure 6).

All participants in Group 3 responded positively to this question, while in Group 1, this indicator was significantly lower (0.029) and reflected 50% of positive responses. In Group 2, this indicator occupied an intermediate value (75% of positive responses) and did not show significant differences with other groups.

## Discussion

The current study found that the quality of life in post-COVID-19 patients is influenced by the severity of the SARS-CoV-2 infection, which is associated with hospitalization and oxygen therapy, as well as outpatient treatment for the SARS-CoV-2 infection. This relationship was shown in the three groups of respondents who received different levels of medical care during the period of COVID-19 disease despite having similar age indicators. A systematic review examined during active COVID-19 described several dozen different symptoms and other quality-of-life issues in patients, ranging from general symptoms (e.g., fever, myalgia, fatigue, and tiredness) to symptoms of neurological and psychological problems and cognitive impairment (Amdal et al., 2021; Hayes et al., 2021). To the best of our knowledge, this study is the first to examine inter-group differences in the quality of life in hospitalized post-COVID-19, who received treatment for varying degrees of severity of the SARS-CoV-2 infection, using a sociodemographic questionnaire, the PSQI, and the EQ-5D-5L test. Hospitalization of SARS-CoV-2-infected patients outside the intensive care unit (not ICU) has had an impact on the quality of life of post-COVID-19 patients in Group 2, even 9 months after recovery. However, according to our data, there was no significant difference in the quality of life between the respondents in Groups 1 and 3. Our study also found that, 6 months after the acute phase of SARS-CoV-2 infection, the quality of life indicators such as “mobility,” “self-care,” “daily activities,” “pain/discomfort,” and “anxiety/depression” did not show any intergroup differences. Therefore, these factors may not be related to the severity of the SARS-CoV-2 infection in the context of the three post-COVID-19 groups. In addition to physical symptoms, people with a post-COVID-19 condition may experience emotional symptoms such as anxiety and depression, which are prevalent during the acute phase of SARS-CoV-2 infection (Li et al., 2021; Liu et al., 2021] and may persist in the post-COVID-19 period (Shanbehzadeh et al., 2021). According to the authors, the presence of physical and emotional symptoms in patients with post-COVID-19 shows that biological and behavioral factors interact in the context of COVID-19 [Hall et al., 2021]. Recent studies have found that higher levels of depressive symptoms are associated with a higher risk of physical symptoms post-COVID-19, such as pain and shortness of breath (Bottemanne et al., 2021].

Our study found that quality of life indicators such as “mobility,” “self-care,” “daily activities,” “pain/discomfort,” and “anxiety/depression” did not show any intergroup differences in the post-COVID-19 period 9 months after the acute phase of SARS-CoV-2 infection and are therefore not related to the severity of the SARS-CoV-2 infection in the context of the three groups of post-COVID-19 conditions considered in our study. However, a subjective assessment of the quality of life on a 100-point scale of the EQ-5D test at 9 months post-COVID-19 revealed significant differences between the respondents who were treated with oxygen therapy during the active phase of the SARS-CoV-2 infection and those who were treated as outpatients. According to Amdal et al. (2021), the number of active COVID-19 publications in the global database of articles was 100 for patients treated in the ICU, 266 for those hospitalized without the ICU, and 49 for those treated in nursing homes, isolation units, or at home. Therefore, we can assume that, after a severe form of infection, SARS-CoV-2-infected patients treated in the ICU at 6 months post-COVID-19 retained the most negative assessment of their quality of life compared to the respondents in Group 2 and Group 3. In a recent study that compared post-COVID-19 symptoms in hospitalized and non-hospitalized patients at 2 years after SARS-CoV-2 infection, no differences in the manifestations of post-COVID-19 were observed (Fernández-de-las-Peñas et al., 2022). According to the authors, this supports the hypothesis that the symptoms post-COVID-19 do not correlate only with the severity of COVID-19. To the best of our knowledge, our study, for the first time, revealed the presence of a relationship at 9 months post-COVID-19 between the quality of life of post-COVID-19 patients and the severity of the SARS-CoV-2 infection in different groups of the hospitalized patients. Previously, other authors suggested that post-COVID-19 affects the daily activity of subjects (Amdal et al., 2021; Pizarro-Pennarolli et al., 2021; Soriano et al., 2022). Our research in the context of analyzing the relationship between the severity of the SARS-CoV-2 infection and the quality of life of post-COVID-19 patients confirms the view that there are more and more data indicating new potential challenges for the health system that long-COVID-19 brings (Menges et al., 2021). It is necessary to emphasize the difference between our study of quality of life indicators at 6 months post-COVID-19 and the abovementioned studies, which examined symptoms detected mainly during the physical examination of respondents, including in conditions of comorbidity. Available evidence suggests that sleep problems are common in people with post-COVID-19 conditions (Iqbal et al., 2021). In a systematic review (Amdal et al., 2021), when describing the symptoms of active COVID-19 function deficits, two reports out of 305 publications showed the problem of insomnia. According to other authors, in the post-COVID-19 phase, sleep quality was disrupted due to the presence of pain symptoms (Pacho-Hernández et al., 2022). In the study by El Sayed et al., it has also been noted that sleep disorders in post-COVID-19 patients are associated with physical and mental aspects of quality of life (El Sayed et al., 2021]. Patients with the post-COVID-19 condition report greater difficulty falling asleep at the desired sleep time and have trouble waking up (Goldstein et al., 2022). Analysis of personal sources on the identified quality problem sleep patterns in the post-COVID-19 period showed that our study is the first comparative study of the sleep quality of respondents with a history of varying severity of the SARS-CoV-2 infection during hospitalization and outpatient treatment at 9 months post-COVID-19. We have established two groups of sleep quality indicators for respondents in three groups. One group of indicators for respondents (sleep duration, sleep disorders, and daytime sleepiness) has a significant relationship with the severity of the SARS-CoV-2 infections. The other group of indicators for sleep quality among respondents (“subjective sleep quality,” “time to sleep,” “sleep efficiency,” and “frequency of taking sleeping pills”) did not demonstrate significant intergroup differences. Consequently, these sleep quality problems appear regardless of the severity of the post-COVID-19 SARS-CoV-2 infection in the 9 months following the acute phase of COVID-19. Thus, our study at 9 months post-COVID-19 confirmed the data from other authors (Amdal et al., 2021; El Sayed et al., 2021; Iqbal et al., 2021; Goldstein et al., 2022; Pacho-Hernández et al., 2022) that the SARS-CoV-2 infection negatively affects sleep quality. The negative impact of a SARS-CoV-2 infection on sleep quality may be the result of a disruption in the circadian regulation system. Moreover, a number of authors have identified the interdependence between circadian disturbances, sleep difficulties, and the COVID-19 pandemic as a major consequence of the COVID-19 crisis on the sleep-wake cycle through lifestyle changes studied in the active stage (1 month) of COVID-19 (Salehinejad et al., 2020, 2022). Notably, a number of indicators of sleep quality are interrelated with the severity of the manifestation of the disease, for which medical oxygen was prescribed to maintain vital signs. This is an important step in understanding the post-clinical manifestation of a previous SARS-CoV-2 infection and its long-term effects on neurophysiological mechanisms such as circadian rhythms during long COVID-19 with differences in the severity of a previous SARS-CoV-2 infection and the prognosis of the disease, as well as its impact on health. The presence of circadian system disorders in the active stage of COVID-19 (Salehinejad et al., 2020) and in the post-COVID-19 stage indicates a long-term disruption in the regulation of circadian biorhythms and the relevance of the rehabilitation of sleep disorders in individuals. It can be assumed that, in the treatment of respondents with post-COVID-19 sleep disorder, circadian technology was not implemented (Pyatin, 2018). In addition, individuals who recovered from COVID-19 had a later chronotype than those without a history of COVID-19 (Han et al., 2023; Tedjasukmana et al., 2023).

Finally, in our context of a comparative study, considering different post-COVID-19 groups by severity of a previous SARS-CoV-2 infection and different medical treatment protocols may be prognostically important, as Rimal et al. (2021) showed in the area of data analysis and visualization, which are essential for exploring and communicating medical research findings, especially when examining COVID-19 records.

## Conclusion

In the study, a total of 123 post-COVID-19 patients who visited SamGMU clinics were includedto. However, only 39 patients (15 men, 24 women) met each of the inclusion criteria; thus, their samples were divided between three groups. For the first time in the history of the SARS-CoV-2 infection, 9 months after the severity of the infection, a quality-of-life assessment (socio-demographic questionnaire, EQ-5D-5L, PAQI) was conducted in two hospitalized groups: those who were treated in oxygen intensive care units (ICU) and those who were treated with anti-COVID-19 therapy. The third group of individuals required outpatient care after being exposed to COVID-19. Although SARS-CoV-2 infection severity differed significantly between the hospitalized post-COVID groups, there was no difference in the quality of life (sociodemographic questionnaire, EQ-5D-5L, PAQI). There was a significant difference between those who were hospitalized and those who were outpatient treated 9 months after hospital discharge in terms of quality of life and sleep disturbances, and there was no difference between groups of patients who were hospitalized.

### Limitations

There are a few limitations to the results of this study, which should be taken into account when interpreting them. The study was conducted in one tertiary care hospital, and the sample size was relatively small; thus, caution should be exercised when disseminating the study results to the general public. Owing to a lack of data available in hospital records, the variables of SARS-CoV-2 in this study did not differ from those observed in previous studies. Sleep quality was assessed using a questionnaire, contributing to the concept of determining the presence of the subjectivity element and the possibility of systematic memory errors being present in the sleep evaluation. For a more critical assessment of the problem of insomnia during prolonged COVID in the long run, higher-quality data may be required to collect complete information. As a result of the assessment of the quality of life, no information was provided regarding the level of assessment that existed prior to the disease's onset. There was no question concerning the average income of the patients that could be found in the sociodemographic questionnaire used by the researchers. It was therefore not possible to assess the impact of this factor on the recovery process as a result of this factor. As the study was conducted online 9 months after discharge from the hospital, one of the questions asked concerning which genetic variant of COVID-19 was more prevalent among the patients was unable to be answered. This study took into account the need to investigate the quality of life indicators with the use of a broader scale of tests in large hospitalized populations to achieve the goals of this project.

## Data availability statement

The raw data supporting the conclusions of this article will be made available by the authors, without undue reservation.

## Ethics statement

The studies involving human participants were reviewed and approved by Ethics Committee of Samara State Medical University. The patients/participants provided their written informed consent to participate in this study.

## Author contributions

Software: AV and SG. Supervision: VP and OM. Project administration: VP, OM, and SG. Conceptualization, validation, investigation, resources, writing—original draft preparation, writing—review and editing, visualization, and methodology: All authors. All authors have read and agreed to the published version of the manuscript.

## References

[B1] Amdal C. D. Pe M. Falk R. S. Piccinin C. Bottomley A. Arraras J. I. (2021). Health related quality of life issues, including symptoms, in patients with active COVID 19 or post COVID 19; a systematic literature review. Qual. Life Res. 30, 3367–3381. 10.1007/s11136-021-02908-z 34146226 PMC8214069

[B2] Belopasov V. V. Zhuravleva E. N. Nugmanova N. P. Abdzashitova A. T. (2021). Post-COVID-19 Neurological Syndromes. J. Clini. Pract. 12, 69–82. 10.17816/clinpract71137

[B3] Bottemanne H. Gouraud C. Hulot J. S. Blanchard A.;, Ranque, B. Lahlou-Laforêt K.. (2021). Do anxiety and depression predict persistent physical symptoms after a severe COVID-19 episode? A prospective study. Front. Psychiatry. 12, 757685. 10.3389/fpsyt.2021.757685 34858230 PMC8631493

[B4] Buysse D. J. Reynolds C. F. Monk T. H. Berman S. R. Kupfer D. J. (1989).The Pittsburgh Sleep Quality Index: a new instrument for psychiatric practice and research. *Psychiat. Res*. 28. 193–213. 10.1016/0165-1781(89)90047-4 2748771

[B5] Carvalho-Schneider C. Laurent E. Lemaignen A. Beaufils E. BourbaoTournois C. Laribi S.. (2021). Follow-up of adults with noncritical COVID-19 two months after symptom onset. Clin. Microbiol. Infect. 27 , 258e263. 10.1016/j.cmi.2020.09.052 33031948 PMC7534895

[B6] CDC HRQOL−14 (2022). Healthy Days Measure. Available online at: https://www.cdc.gov/hrqol/hrqol14_measure.htm (accessed Oct 2022).

[B7] Chen C. Haupert S. R. Zimmermann L. Shi X. Fritsche L. G. Mukherjee B. (2022). Global prevalence post COVID-19 condition or long COVID: a meta-analysis and systematic review. J. Infect. Dis. 16. 10.1101/2021.11.15.21266377 35429399 PMC9047189

[B8] Del Rio C. Collins L. F. Malani P. (2020). Long-term health consequences of COVID-19. JAMA. 324, e1724. 10.1001/jama.2020.19719 33031513 PMC8019677

[B9] El Sayed S. Gomaa S. Shokry D. Kabil A. Eissa A. (2021). Sleep in postCOVID-19 recovery period and its impact on different domains of quality of life. Egypt. J. Neurol. Psychiatr. Neurosurg. 57, 172. 10.1186/s41983-021-00429-7 34924750 PMC8669420

[B10] Feng Y. Parkin D. Devlin N. J. (2014). Assessing the performance of the EQ VAS in the NHS PROMs programme. Qual. Life Res. 23, 977–989. 10.1007/s11136-013-0537-z 24081873 PMC4287662

[B11] Feng Y. S. Kohlmann T. Janssen M. F. Buchholz I. (2021). Psychometric properties of the EQ-5D-5L: a systematic review of the literature. Qual Life Res. 30, 647–673. 10.1007/s11136-020-02688-y 33284428 PMC7952346

[B12] Fernández-de-las-Peñas C. Rodríguez-Jiménez J. Cancela-Cilleruelo I. Guerrero-Peral A. Martín-Guerrero J. D. García-Azorín D.. (2022). Post–COVID-19 symptoms 2 years after SARS-CoV-2 infection among hospitalized vs. nonhospitalized patients. JAMA Network Open. 5 , e2242106. 10.1001/jamanetworkopen.2022.42106 36378309 PMC9667330

[B13] Goldstein C. A. Kagan D. Rizvydeen M. Warshaw S. Troost J. P. Burgess H. J. (2022). The possibility of circadian rhythm disruption in long COVID. Brain Behave. Immun. Health. 23, 100476. 10.1016/j.bbih.2022.100476 35663839 PMC9153185

[B14] Hall P. A. Sheeran P. Fong G. T. Cheah C. S. L. Oremus M. LiuAmbrose T.. (2021). Biobehavioral aspects of the COVID-19 pandemic: a review. Psychosom. Med. 83, 309–321. 10.1097/PSY.0000000000000932 33790201 PMC8115744

[B15] Han S. H. Lee S. Y. Cho J. W. Kim J. H. Moon H. J. Park H. R. Cho Y. W. (2023). Sleep and circadian rhythm in relation to COVID-19 and COVID-19 vaccination—national sleep survey of South Korea 2022. J. Clin. Med. 12, 1518. 10.3390/jcm12041518 36836053 PMC9967239

[B16] Hawthorne G. Richardson J. Day N. A. (2001). A comparison of the Assessment of Quality of Life (AQoL) with four other generic utility instruments. Ann. Med. 33, 358–370. 10.3109/07853890109002090 11491195

[B17] Hayes L. D. Ingram J. Sculthorpe N. F. (2021). More than 100 persistent symptoms of SARS-CoV-2 (long COVID): a scoping review. Front, Med. 8. 750378. 10.3389/fmed.2021.750378 34790680 PMC8591053

[B18] Iqbal F. M. Lam K. Sounderajah V. Clarke J. M. Ashrafian H. Darzi A. (2021). Characteristics and predictors of acute and chronic post-COVID syndrome: a systematic review and meta-analysis. EClinical Med. 36, 100899. 10.1016/j.eclinm.2021.100899 34036253 PMC8141371

[B19] Karimi M. Brazier J. (2016). Health, health-related quality of life, and quality of life: what is the difference? Pharmacoeconomics. 34, 645–649. 10.1007/s40273-016-0389-9 26892973

[B20] Kaul M. Gupta P. Kalra S. Gardner J. Gordon H. S. Rubinstein I. (2022). New domiciliary supplemental oxygen therapy after hospitalisation for COVID-19 in metropolitan Chicago. ERJ Open Res. 8 , 00577–2021. 10.1183/23120541.00577-2021 35345420 PMC8883040

[B21] Li T. Sun S. Liu B. Wang J. Zhang Y. Gong C. Duan J. (2021). Prevalence and risk factors for anxiety and depression in patients with COVID-19 in Wuhan, China. Psychosom. Med. 83, 368–372. 10.1097/PSY.0000000000000934 33951724

[B22] Liu C. Pan W. Li L. Li B. Ren Y. Ma X. (2021). Prevalence of depression, anxiety, and insomnia symptoms among patients with COVID-19: a metaanalysis of quality effects model. J. Psychosom. Res. 147, 110516. 10.1016/j.jpsychores.2021.110516 34023580 PMC8129994

[B23] Lopez-Leon S. Wegman-Ostrosky T. Perelman C. Sepulveda R. Rebolledo P. A. Cuapio A. Villapol S. (2021). More than 50 long-term effects of COVID-19: a systematic review and meta-analysis. Sci. Reports. 11, 6144. 10.1038/s41598-021-95565-8 34373540 PMC8352980

[B24] Luca G. Haba Rubio J. Andries D. Tobback N. Vollenweider P. Waeber G. (2015). Age and gender variations of sleep in subjects without sleep disorders. Ann. Med. 47, 482–491. 10.3109/07853890.2015.1074271 26224201

[B25] Menges D. Ballouz T. Anagnostopoulos A. Aschmann H. E. Domenghino A. Fehr J. S. Puhan M. A. (2021). Burden of post-COVID-19 syndrome and implications for healthcare service planning: a population-based cohort study. PLoS ONE. 16, e0254523. 10.1371/journal.pone.0254523 34252157 PMC8274847

[B26] Michelen M. Manoharan L. Elkheir N. Cheng V. Dagens A. Hastie C.. (2021). Characterising long COVID: a living systematic review. BMJ Global Health. 6, e005427. 10.1136/bmjgh-2021-005427 34580069 PMC8478580

[B27] Nandasena H. M. R. K.G. Pathirathna M. L. Atapattu A. M. M. P. Prasanga P. T. S. (2022). Quality of life of COVID 19 patients after discharge: systematic review. PLoS ONE. 17, 1–12. 10.1371/journal.pone.0263941 35171956 PMC8849513

[B28] Nasserie T. Hittle M. Goodman S. N. (2021). Assessment of the frequency and variety of persistent symptoms among patients. COVID-19: a systematic review. JAMA Netw. Open. 4, e2111417. 10.1001/jamanetworkopen.2021.11417 34037731 PMC8155823

[B29] Pacho-Hernández J. C. Fernández-de-Las-Peñas C. Fuensalida-Novo S. Jiménez-Antona C. Ortega-Santiago R. Cigarán-Mendez M. (2022). Sleep quality mediates the effect of sensitization-associated symptoms, anxiety, and depression on quality of life in individuals with post-covid-19 pain. Brain Sci. 12, 1363. 10.3390/brainsci12101363 36291297 PMC9599807

[B30] Pazukhina E. Andreeva M. Spiridonova E. Bobkova P. Shikhaleva A. El-Taravi Y (2022). Prevalence and risk factors of post-COVID-19 condition in adults and children at 6 and 12 months after hospital discharge: a prospective, cohort study in Moscow (StopCOVID). BMC Med. 20, 244. 10.1186/s12916-022-02448-4 35794549 PMC9257572

[B31] Peghin M. Palese A. Venturini M. De Martino M Gerussi V. Graziano E.. (2021). Post-COVID-19 symptoms 6 months after acute infection among hospitalized and non-hospitalized patients. Clin. Microbiol. Infect. 27, 1507–1513. 10.1016/j.cmi.2021.05.033 34111579 PMC8180450

[B32] Pérez-González A. Araújo-Ameijeiras A. Fernández-Villar A. Crespo M. Poveda E. (2022). Cohort COVID-19 of the Galicia Sur Health Research Institute. Long COVID in hospitalized and non-hospitalized patients in a large cohort in Northwest Spain, a prospective cohort study. Sci. Rep. 12, 3369. 10.1038/s41598-022-18023-z 35233035 PMC8888560

[B33] Phua J. Weng L. Ling L. Egi M. Lim C. M. Divatia J. V.. (2020). Intensive care management of coronavirus disease 2019 (COVID-19): challenges and recommendations. Lancet Respir. Med. 8, 506–517. 10.1016/S2213-2600(20)30161-2 32272080 PMC7198848

[B34] Pizarro-Pennarolli C. Sánchez-Rojas C. Torres-Castro R. Vera-Uribe R. Sanchez-Ramirez D. C. Vasconcello-Castillo L.. (2021). Assessment of activities of daily living in patients post COVID-19: a systematic review. Peer J. 9, e11026. 10.7717/peerj.11026 33868804 PMC8034364

[B35] Pomara C. Li Volti G. Cappello F. (2020). The post-lockdown era: what is next in Italy? Front. Pharmacol. 11, 1074. 10.3389/fphar.2020.01074 32760280 PMC7373777

[B36] Poude A. N. Zhu S. Coope N. Roderick P. Alwan N. Tarrant C.. (2021). Impact of Covid-19 on health-related quality of life of patients: a structured review. PLoS One. Oct.28. 16(10):e0259164. 10.1371/journal.pone.0259164 34710173 PMC8553121

[B37] Pyatin V. F. (2018). Device for Functional Control of Human Body Circadian Clock. RUSSIAN patent No. 182615. Bulletin No.24. Federal State Budget Institution Federal Institute of Industrial Property, Moscow, Russia.

[B38] RAND Corporation (2022). 36-Item Short Form Survey (SF-36). Available online at: https://www.rand.org/health-care/surveys_tools/mos/36-item-short-form.html (accessed 23 January 2022).

[B39] Rimal Y. Gochhait S. Bisht A. (2021). Data interpretation and visualization of COVID-19 cases using R programming. Inf. Med. Unlocked. 26, 100705. 10.1016/j.imu.2021.100705 34485681 PMC8404394

[B40] Saadatmand S. Salimifard K. Mohammadi R. Marzban M Naghibzadeh-Tahami A. (2022). Predicting the necessity of oxygen therapy in the early stage of COVID-19 using machine learning. Med. Biol. Eng. Comput. 60, 957–968. 10.1007/s11517-022-02519-x 35147843 PMC8832434

[B41] Salehinejad M. A. Azarkolah A. Ghanavat,i E. Nitsche M.A. (2022). Circadian disturbances, sleep difficulties and the COVID-19 pandemic. Sleep Med. 91, 246e252. 10.1016/j.sleep.2021.07.011 34334305 PMC8277544

[B42] Salehinejad M. A. Majidinezhad M. Ghanavati E. Kouestanian S. Vicario C. M. Nitsche M. A. Nejati V. (2020). Negative impact of COVID-19 pandemic on sleep quantitative parameters, quality, and circadian alignment: implications for health and psychological well-being. EXCLI J. 19, e308. 10.1101/2020.07.09.20149138 33192213 PMC7658458

[B43] Shanbehzadeh S. Tavahomi M. Zanjari N. Ebrahimi-Takamjani I. Amiri-Arimi S. (2021). Physical and mental health complications post-COVID-19: Scoping review. J. Psychosom. Res. 147, 110525. 10.1016/j.jpsychores.2021.110525 34051516 PMC8133797

[B44] Sivan M. Parkin A. Makower S. Greenwood D. C. (2022). Post-COVID syndrome symptoms, functional disability, and clinical severity phenotypes in hospitalized and non hospitalized individuals: a cross-sectional evaluation from a community COVID rehabilitation service. J. Med. Virol. 94, 1419–1427. 10.1002/jmv.27456 34783052 PMC8661751

[B45] Soriano J. B. Murthy S. Marshall J. C. Relan P. Diaz J. V. (2022). WHO clinical case definition working group on PostCOVID-19 condition. A clinical case definition of post-COVID-19 condition by a Delphi consensus. Lancet. Infect Dis. 22, e102–e107. 10.1016/S1473-3099(21)00703-9 34951953 PMC8691845

[B46] Tedjasukmana R. Budikayanti A. Islamiyah W. R. Witjaksono A. M. A. L. Hakim M. (2023). Sleep disturbance in post COVID-19 conditions: prevalence and quality of life. Front Neurol. Jan. 9, 13:1095606. 10.3389/fneur.2022.1095606 36698905 PMC9869804

[B47] Van Kessel S. A. M. Olde Hartman T. C. Lucassen P. L. B. J. van Jaarsveld C. H. M. (2022). Post-acute and long–COVID-19 symptoms in patients with mild diseases: a systematic review. Fam. Pract. 39, 159–167. 10.1093/fampra/cmab076 34268556 PMC8414057

